# Clinicopathological correlations in heart transplantation recipients complicated by death or re-transplantation

**DOI:** 10.3389/fcvm.2022.1014796

**Published:** 2022-11-03

**Authors:** Michelle M. McDonald, Maks Mihalj, Bihong Zhao, Sriram Nathan, Stanislava Matejin, Giulia Ottaviani, Mateja K. Jezovnik, Rajko Radovancevic, Biswajit Kar, Igor D. Gregoric, L. Maximilian Buja

**Affiliations:** ^1^Department of Pathology and Laboratory Medicine, University of Texas Health Science Center at Houston, Houston, TX, United States; ^2^Department of Advanced Cardiopulmonary Therapies and Transplantation, University of Texas Health Science Center at Houston, Houston, TX, United States; ^3^Department of Cardiac Surgery, Bern University Hospital, University of Bern, Bern, Switzerland; ^4^Cardiovascular Pathology, Lino Rossi Research Center, Department of Biomedical, Surgical and Dental Sciences, Università degli Studi di Milano, Milan, Italy

**Keywords:** heart transplantation, endomyocardial biopsy, acute myocardial injury, primary graft dysfunction, rejection, cardiac allograft vasculopathy, C4d

## Abstract

**Purpose:**

This study aimed to identify and correlate pathological findings with clinical outcomes in patients after orthotopic heart transplantation (OHT) who either died or underwent a re-transplantation.

**Methodology and study design:**

Single-center retrospective analysis of primary OHT patients who died or were re-transplanted between October 2012 and July 2021. Clinical data were matched with corresponding pathological findings from endomyocardial biopsies on antibody-mediated rejection, cellular rejection, and cardiac allograft vasculopathy. Re-assessment of available tissue samples was performed to investigate acute myocardial injury (AMI) as a distinct phenomenon. These were correlated with clinical outcomes, which included severe primary graft dysfunction. Patients were grouped according to the presence of AMI and compared.

**Results:**

We identified 47 patients with truncated outcomes after the first OHT. The median age was 59 years, 36 patients (76%) were male, 25 patients (53%) had a prior history of cardiac operation, and 21 patients (45%) were supported with a durable assist device before OHT. Of those, AMI was identified in 22 (47%) patients (AMI group), and 25 patients had no AMI (non-AMI group). Groups were comparable in baseline and perioperative data. Histopathological observations in AMI group included a non-significant higher incidence of antibody-mediated rejection Grade 1 or higher (pAMR ≥ 1) (32% vs. 12%, *P* = 0.154), and non-significant lower incidence of severe acute cellular rejection (ACR ≥ 2R) (32% vs. 40%, *P* = 0.762). Clinical observations in the AMI group found a significantly higher occurrence of severe primary graft dysfunction (68% vs. 20%, *P* = 0.001) and a highly significant shorter duration from transplantation to death or re-transplantation (42 days [IQR 26, 120] vs. 1,133 days [711–1,664], *P* < 0.0001). Those patients had a significantly higher occurrence of cardiac-related deaths (64% vs. 24%, *P* = 0.020). No difference was observed in other outcomes.

**Conclusion:**

In heart transplant recipients with a truncated postoperative course leading to either death or re-transplantation, AMI in endomyocardial biopsies was a common pathological phenomenon, which correlated with the clinical occurrence of severe primary graft dysfunction. Those patients had significantly shorter survival times and higher cardiac-related deaths. The presence of AMI suggests a truncated course after OHT.

## Introduction

Advanced heart failure (HF) remains a detrimental condition associated with high mortality. While alternative strategies using mechanical circulatory support devices (MCS) may offer durable support for those patients, either before transplantation or as destination therapy, they are associated with a high comorbidity profile (1–4). Overall, orthotopic cardiac transplantation (OHT) remains the best therapeutic option to improve long-term survival and quality of life in patients with advanced HF (5–7). The current median survival time for adult patients after OHT is 11.5 years, with a contingent survival of 13.9 years for those who survive after the first year (5–7). Nevertheless, OHT recipients are exposed to the risk of several potential complications that may impair their outcomes (8, 9). These include graft-related complications, which include primary graft dysfunction (PGD), acute cellular rejection (ACR), antibody-mediated rejection (AMR), and cardiac allograft vasculopathy (CAV). Non-graft-related complications also impact transplant patients, including infections, renal dysfunction, and malignancy. While late mortality is commonly associated with the latter and with CAV, early mortality is often dominated by PGD and ACR (10–12). Specific donor-, recipient-, and surgery-related risk factors have been associated with PGD (9, 13), but the pathophysiological mechanisms remain largely unknown (9–12, 14–17). Standardized endomyocardial biopsies (EMB) are performed to detect rejection and adjust immunosuppressive therapy, but pathological changes associated with PGD are not routinely investigated in EMB samples. Further, no reliable marker exists to detect PGD to this date. Importantly, contraction band necrosis as a sign of acute myocardial injury (AMI) has been previously described in donor hearts supported with high inotropic support after brain death (9, 18–23). Still, a knowledge gap remains in whether these findings correlate with worse clinical outcomes. This study aimed to correlate the pathological findings in EMB with clinical outcomes of those OHT recipients who either died or underwent a re-transplantation.

## Materials and methods

### Study design

The observational and retrospective analysis included all adult patients who underwent a primary OHT at our tertiary care institution between October 2012 and July 2021. Only those OHT recipients were included who had a “hard outcome,” defined as either postoperative death or re-OHT, whichever occurred first. Patients were followed up until death, re-OHT, or until July 31, 2021 (censor date). EMB biopsies were performed per our institutional protocol in every OHT recipient regardless of clinical status, at the end of weeks 1, 2, 3, 4, 6, 8, and 12, as well as at 6 months and optionally at 9 months after OHT, based on rejection history, level of immunosuppression and results of Allomap^®^ and Allosure^®^ assays for gene mapping and donor-derived cell-free DNA. For patients exhibiting adverse clinical features, the first EMB may be done sooner and repeat EMBs may be performed more frequently, which is considered in the time to event analysis. Upon de-identifying the included patients, their corresponding stored tissue samples were re-analyzed for rejection and stained additionally for the occurrence of cardiomyocyte (CMC) AMI necrosis (see below).

**FIGURE 1:**
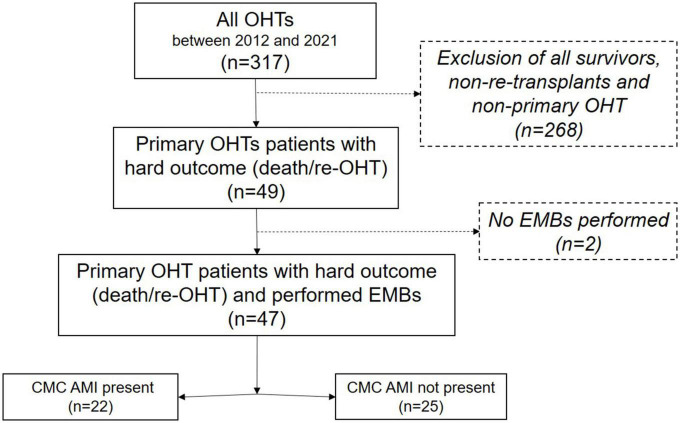
Study flow chart. The patient population is included in this retrospective review, and their interventions and outcomes are summarized in this flow chart. OHT, orthotopic heart transplantation; CMC, cardiomyocyte; AMI, acute myocardial injury; EMB, endomyocardial biopsy.

Further exploratory outcomes included histopathological signs of Grade ≥ 1 AMR, Grade ≥ 2 ACR, and CAV obtained from either EMB or an autopsy. All available EMB samples and tissue samples from the autopsy were analyzed. The clinical outcomes included peri-/postoperative extracorporeal membrane oxygenator (ECMO) support (differentiating between immediate ECMO support referring to intraoperative EMCO installment during index surgery, and delayed ECMO support, referring to postoperative ECMO installment), hospitalization duration after transplant, and the clinical occurrence of severe PGD, as defined by Kobashigawa et al. (9), until the hard outcomes of death or re-transplantation. Patient data included demographics, pre- and perioperative characteristics, and postoperative outcomes and was collected from internal electronic medical records by trained study personnel in an anonymized way. Patients were grouped according to the presence of CMC AMI in EMB (“AMI group” vs. “Non-AMI group”). This is summarized in the study flow chart (Figure 1). The study was conducted under the principles outlined in the Declaration of Helsinki and was approved by the institutional review board (HSC-MS-14-0139).

### Pathology evaluation

All pathology specimens were identified from the electronic medical records, and two experienced cardiac pathologists (MM and LMB) performed a *de novo* pathological re-examination on all available tissue samples. The tissue samples were de-identified and anonymized, and the pathologist was blinded to the prior clinical and pathology reports during re-examination. The pathology specimens included tissue samples from all available post-OHT EMBs and donor transplant hearts at autopsy. EMB findings are presented regarding evidence of AMR and ACR in the standardized nomenclature (24, 25) and evidence of CMC AMI. The tissue samples were evaluated for the following histological types of AMI (26–28):

(i) hydropic/vacuolar change (moderate-sized, fluid-filled vacuoles);
(ii) fatty change (fine vacuoles containing triglycerides);
(iii) myocytolysis (colliquative myocytolysis) (swelling with loss of myofibrils);
(iv) contraction band injury/necrosis (coagulative myocytolysis);
(v) and coagulation necrosis (acontractile necrosis).

Only EMBs with the highest grades of injury and antibody-mediated and/or cellular rejection were reported. Evidence of AMI was also evaluated based on immunohistochemical detection with C4d (29). Therefore, a subgroup analysis was performed on a subset of those using C4d staining to confirm the histological findings and correlate C4d IHC with the H&E evidence of damage. It is important to note here that the pattern of C4d IHC staining in identifying acute cardiomyocyte damage is completely different from the pattern of staining associated with AMR. In C4d IHC, the marker is taken up by the CMC and does not highlight the capillaries as in C4d staining in AMR diagnostics.

### Statistical analysis

The continuous or discrete data distribution was assessed with Shapiro–Wilk tests, with most variables showing a skewed distribution. Therefore, we consistently report medians and quartiles for continuous data and used non-parametric testing (Mann–Whitney *U* tests) for comparison between groups. Categorical data are summarized as counts and percentages and were compared between groups using Fisher’s exact tests. Survival analysis techniques were used for time-to-event data; specifically, Kaplan–Meier curves and log-rank tests were used to compare time-to-first event between the groups for comparison between groups for hard outcomes, as well as for the occurrence of worse documented rejection. The selected level of significance was *P* < 0.05, two-tailed. All statistical analyses were performed in Stata/IC 16.0 (StataCorp., College Station, TX, USA).

## Results

Primary OHT was performed in 317 patients within the study observation period. Of these, 49 OHT recipients (15.5%) were identified who had either died or had to undergo a re-transplantation. Two were excluded due to missing EMB samples (4.1%).

The remaining 47 patients were evaluated for the distribution of pathological observations. The median age of these 47 patients was 59 years [interquartile range (IQR) 51, 67]. Thirty-six patients (76%) were male, with a median BMI of 28 kg/m^2^ (23, 32). In summary, ischemic cardiomyopathy was present in 23 patients (49%) prior to OHT, and the most common comorbidities were arterial hypertension and dyslipidemia (65 and 51%, respectively). Twenty-five patients (53%) had a prior history of cardiac operation, and 21 patients (45%) were supported with a left ventricular assist device (LVAD) or total artificial heart (TAH) before OHT.

We found in EMB that CMC AMI was present in 22 (46.8%) patients and absent in 25 (53.2%). Patients were therefore grouped according to the presence or absence of CMC AMI. No significant differences were observed in any of the investigated demographic data between the groups (Table 1).

**TABLE 1:** Baseline demographics and perioperative data.

Variable	All patients (*N* = 47)	Patients with AMI EMB (*N* = 22)	Patients without AMI (*N* = 25)	*P*-value
**Demographics**				
Age (years)	59 (51, 67)	56 (51, 63)	66 (50, 68)	0.290
BMI (kg/m^2^)	28 (23, 32)	30.5 (28, 33)	26 (23, 31)	0.420
BSA (m^2^)	1.98 (1.77, 2.25)	2.02 (1.82, 2.27)	1.98 (1.76-2.23)	0.655
Sex, male	36 (76%)	16 (73%)	20 (80%)	0.732
Race				0.264
White	19 (40%)	6 (27%)	13 (52%)	
African-American	16 (34%)	10 (45%)	6 (24%)	
Hispanic	8 (17%)	4 (18%)	4 (16%)	
Asian	3 (6%)	1 (4%)	2 (8%)	
Not disclosed	1 (2%)	1 (4%)	0	
Ischemic cardiomyopathy	23 (49%)	10 (45%)	13 (52%)	0.772
Arterial hypertension	30 (65%)	13 (61%)	17 (68%)	0.750
Diabetes mellitus	18 (39%)	10 (44%)	8 (36%)	0.753
Dyslipidemia	24 (51%)	10 (44%)	14 (56%)	0.543
Atrial fibrillation	12 (28%)	5 (28%)	7 (28%)	1.000
History of CVI	4 (9%)	3 (17%)	1 (4%)	0.293
Re-sternotomy	25 (53%)	14 (64%)	11 (44%)	0.244
MCS device before transplantation	21 (45%)	11 (50%)	10 (40%)	0.564
**Perioperative data**				
Total ischemic time (min)	202 (153, 222)	203 (170, 218)	174 (105, 225)	0.177
CPB time (min)	127 (110, 153)	145 (121, 182)	123 (109, 137)	0.115
Immediate ECMO post OHT	8 (17%)	6 (27%)	2 (8%)	0.123
Delayed ECMO post OHT	18 (38%)	12 (55%)	6 (24%)	0.040
Duration of ECMO support (days)	10 (6, 21)	10 (4, 21)	11 (7, 33)	0.413

All data as median (IQR) or counts (%) BMI, body mass index; BSA, body surface area; ICD, implantable cardioverter defibrillator; CVI, cerebrovascular insult; AMI, acute myocardial injury; EMB, endomyocardial biopsy; CPB, cardiopulmonary bypass; MCS, mechanical circulatory support; ECMO, extracorporeal membrane oxygenation.

Perioperatively, no significant differences were observed between the groups except for the need for delayed ECMO support in the AMI necrosis group (55% vs. 24%; *P* = 0.04). The median allograft ischemic time was 203 min (170, 218) in the AMI group, and 174 min (105, 225) in the non-AMI group; although this difference was not significant (*P* = 0.177). The postoperative median duration of ECMO support was 10 days (6, 21) and was non-significant between groups.

### Clinical outcomes

**FIGURE 2:**
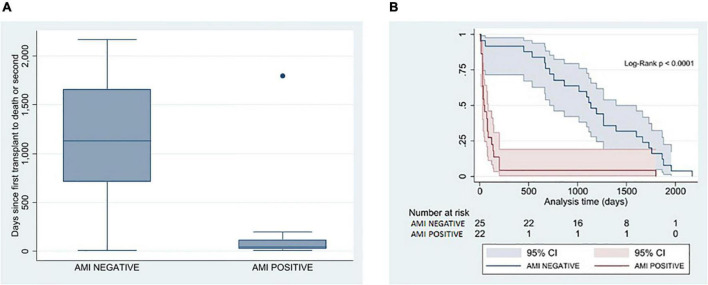
Correlation between pathological presence of AMI in EMB and time from the first transplant to death or re-transplant. **(A)** Box plot analysis between AMI and non-AMI patients on time from the first transplant to death or re-transplant in days. **(B)** Kaplan–Meier estimator on freedom-from hard outcome death or re-transplantation between the AMI (red) and non-AMI patients (blue). AMI, acute myocardial injury; EMB, endomyocardial biopsy.

Altogether, six patients (13%) underwent a re-OHT; the cases were evenly divided between groups. The remaining 41 patients died (87%). Patients with CMC AMI had a significantly shorter time from OHT to death or re-OHT (42 days [26, 120] vs. 1,133 days [711, 1,664], *P* < 0.0001) (Table 2 and Figure 2). The clinical course of patients with documented AMI in EMB was dominated by a significantly higher occurrence of severe PGD. This was observed in 15 patients (68%), compared to five patients (20%) where AMI was not observed (*P* = 0.001). The most common cause of death in the AMI group was cardiac-related death (14/22, 64% vs. 6/25, 24%), whereas non-cardiac-related death (causes due to multiorgan failure, sepsis, cerebrovascular event, cancer, or unknown causes) was significantly more common in patients without AMI (27% vs. 64%; *P* = 0.020).

**TABLE 2:** Clinicopathological outcomes.

Variable	All patients (*N* = 47)	Patients with AMI (*N* = 22)	Patients without AMI (*N* = 25)	*P*-value
**Clinical outcomes**				
Severe PGD	20 (42%)	15 (68%)	5 (20%)	0.001
Re-OHT	6 (13%)	3 (14%)	3 (12%)	1.000
Cause of Death				0.020
Cardiac	21 (45%)	14 (64%)	6 (24%)	
Non-cardiac	20 (43%)	5 (23%)	16 (64%)	
Time from OHT to death/re-OHT (days)	446 (38, 1264)	42 (26, 120)	1133 (711, 1664)	< 0.0001
**Pathological outcomes**				
EMB performed	47 (100%)	22 (100%)	25 (100%)	1
C4d Staining of CMC	21 (45%)	15/20 (75%)	0/1 (0%)	0.154
Worst Grade ≥ 1 AMR	10 (21%)	7 (32%)	3 (12%)	0.154
Median time to worst grade ≥ 1 AMR (days)	24 (17, 163)	22 (8, 28)	1157 (23, 1392)	0.087
Worst Grade ≥ 2 ACR	17 (36%)	7 (32%)	10 (40%)	0.762
Median time to worst grade ≥ 2 ACR (days)	20 (15, 61)	16 (7, 31)	56 (15, 179)	0.064
CAV	6 (13%)	1 (5%)	5 (20%)	0.194

All data as median (IQR) or counts (%) AMI, acute myocardial injury; PGD, primary graft dysfunction; OHT, orthotopic heart transplantation; EMB, endomyocardial biopsy; CMC, cardiomyocyte; AMR, antibody-mediated rejection; ACR, acute cellular rejection; CAV, cardiac allograft vasculopathy.

### Pathological findings

**FIGURE 3:**
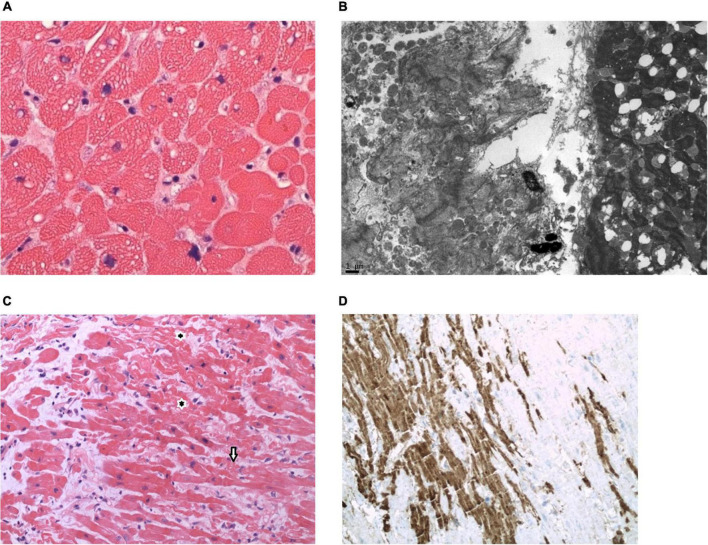
Pathological observations. **(A,B)**. Endomyocardial biopsy (EMB) on post-orthotopic heart transplant (OHT) Day 2 from a 55-year-old man with non-ischemic cardiomyopathy associated with cardiac sarcoidosis who manifested clinical features of early graft dysfunction. Triglyceride droplets in cardiomyocytes (CMC) are evidence of CMC injury. EMB confirmed the lipidosis and focal contraction band necrosis of CMC. Patient expired on post-OHT Day 28. **(C,D)**. EMB on post-OHT Day 5 from a 71-year-old man with non-ischemic cardiomyopathy who manifest clinical features of early graft dysfunction. EMB shows features of CMC injury with contraction bands (arrows) and marked C4d uptake into the damaged CMC. Patient expired on post-OHT day 35. [**(A)**, hematoxylin and eosin stain, high magnification; **(B)**, electron micrograph; **(C)**, hematoxylin and eosin stain, medium magnification; **(D)**, C4d immunostain, medium magnification].

The CMC AMI was characterized by isolated coagulation necrosis in nine patients (41%), isolated vacuolar change in three patients (14%), isolated myocytolysis in two patients (9%), isolated contraction band injury in two patients (9%), coagulative necrosis with vacuolar change in two patients (9%), myocytolysis with vacuolar change in one patient (5%), contraction band injury with vacuolar change in one patient (5%), a combination of contraction band necrosis and fatty change in one patient (5%; Figures 3A,B), and myocytolysis with contraction band injury in one patient (5%).

Antibody-mediated rejection of grade pAMR 1 or 2 was observed in ten patients (21%), of which seven (32%) were found in the AMI group and three (12%) in the non-AMI group. No cases had a pAMR 3 grade. The median time to the first observation of the respective pAMR 1 or pAMR 2 in patients with documented AMI was 22 days (8, 28). In contrast, it was observed after a median of 1157 days (23, 1,392) in the three patients without AMI (*P* = 0.087). Severe ACR of grade 2R or 3R was observed in 17 patients (36%) but without evidence of a significant difference in the occurrence of severe ACR between groups (*P* = 0.762). The median time to severe ACR was 16 days (7, 31) for the AMI group and 56 days (15, 179) for the non-AMI group (*P* = 0.064). No significant difference was observed in the occurrence of CAV, which was observed in only one patient with documented AMI (5%), and in five patients (20%) in the non-AMI group (*P* = 0.194).

To evaluate the utility of C4d IHC in identifying AMI, 51 biopsies from 21 patients with hard outcomes in the first year after transplant were selected for C4d staining. The biopsies selected for staining were taken within the first two months after OHT. Of the 51 biopsies selected, twenty-eight (55%) had evidence of AMI by routine hematoxylin and eosin (H&E) staining. Focal C4d staining was found in 64.3% (18/28) of the biopsies with AMI damage, and only one biopsy (3.6%) without damage showed minimal focal C4d staining. In looking at the types of damage associated with C4d uptake, we noted six biopsies had only vacuolar change, five of which did not show C4d staining. The remaining 22 biopsies had more severe damage, and 17 of these biopsies showed C4d staining (Figures 3C,D). We also noted five biopsies with contraction bands, without necrosis—all of which showed C4d staining. In summary, of the 22 identified patients (45%) with AMI, C4d staining was performed in 21 patients, of which 15 (71%) had one or more biopsies taken during the first two months post OHT, which stained positive for C4d.

CAV was observed in one patient with detected CMC AMI (5%), who died 73 days after OHT, and in five patients where no CMC AMI was detected (20%). These patents died or were re-transplanted after median 711 days (598, 1409).

### Correlation between severe primary graft dysfunction and cardiomyocyte acute myocardial injury

Out of the 22 patients with detected CMC AMI in EMB, 15 patients (68.2%) had severe PGD and only seven (31.8%) had no evidence of severe PGD. Correspondingly, the remaining 25 patients did not have evidence for CMC AMI in EMB, and only five of those (20%) had evidence of severe PGD. The absence of CMC AMI in EMB had a high negative predictive value of 80% (95% CI 68–91), relatively high sensitivity of 75% (95% CI 62–87), and specificity of 74% (62–87) for severe PGD. A significantly higher occurrence of CMC AMI was observed in those who died within the first 90 days after OHT [88.9% (16/18) of patients vs. 20.7% (6/29) of patients; *P* < 0.0001]. Similarly, a significantly higher occurrence of severe PGD was observed in those who died within the first 90 days after OHT [77.8% (14/18) of patients vs. 20.7% (6/29) of patients; *P* = 0.0002].

## Discussion

Our findings document that post-OHT EMB can provide evidence of CMC AMI, which can indicate primary graft dysfunction based on the strong correlation obtained from our data. We showed a spectrum of AMI injury types and severity seen in the biopsies. We also showed that the histopathological features correlated with diffuse C4d deposition in the injured CMC.

The C4d deposition is a consequence of sarcolemmal damage, a key component of myocardial ischemic injury (10, 11, 30). Manzoor and colleagues reported similar results regarding EMB findings in patients with PGD (23). Their study included 20 PGD EMBs, and 50% (10/20) showed myofiber injury/necrosis by either morphology and/or C4d/C3d IHC. One case had ACR (grade 1R, ISHLT 2004), and two had AMR 2 (ISHLT 2013). In a control group of 24 cases, 5 showed myofiber injury, 3 had ACR (grade 1R, ISHLT 2004), and 2 had AMR 2 (ISHLT 2013). Manzoor and colleagues concluded that myofiber injury, including coagulative necrosis, are the pathologic features of severe cardiac PGD. Their findings support AMI as a separate etiology and do not indicate ACR or AMR involvement. A similar observation was made in an older study by Baldwin et al., where they found C4d depositions in pericapillary regions in EMBs obtained within three weeks of transplantation in 15 (45%) of the 33 patients (31). Histopathological evidence of myocardial ischemic injury was detected in 11 (73%) of the 15 biopsies with C4d and/or C3d deposition, compared to 8 (44%) biopsies without C4d and/or C3d deposition (P = 0.005). This supports our findings of AMI as a separate entity, independent of ACR and AMR.

Regarding the clinical outcomes, the patients with detected CMC AMI had a significantly higher occurrence of severe PGD. Furthermore, patients with severe PGD had a significantly shorter survival time or time until re-transplantation. The findings of this study may suggest that CMC AMI may be a marker for pathological changes leading to PGD. Further, CMC AMI may be used as a predictor of early mortality in OHT recipients with a truncated outcome.

Cardiac transplantation is a clinical setting that involves risk for global myocardial ischemia during harvest, transport, and implantation of the donor heart (10–12). Techniques of cardioplegia derived from open heart surgery have been adapted to protect the donor heart. Nevertheless, myocardial ischemia and cardiac reperfusion injury can be major factors in the development of early graft dysfunction shortly after implantation of the donor heart (9, 32–37). It has been shown that the total myocardial ischemic time of four hours in the conventional static preservation methods is associated with significantly impaired overall survival (38, 39). However, this time may be significantly prolonged with novel procurement strategies with continuous *ex vivo* perfusion (36, 40–42). All of the “failed” OHT recipients in our study had an ischemic time kept under 240 min; however, non-significant longer ischemic times were observed in the AMI group (median 29 min longer ischemia time). While this difference is not significant, it is relevant since all baseline characteristics between groups were comparable. Nonetheless, patients in the AMI group had a higher incidence of ECMO support after their OHT, a higher incidence of PGD, and significantly shorter survival times. Therefore, the importance of graft ischemic time of <180 min in the non-AMI group on PGD and overall clinical outcome should be evaluated in larger studies and further examined.

This combination of ischemia and myocardial reperfusion injury is perhaps the main contributor to altered short-term outcomes after OHT, including PGD. The conventional static preservation procurement strategies aim at reducing the preservation injury during cold ischemia. The main objectives of organ preservation are to establish hypothermia, prevent cell swelling, and minimize free radical-induced organ injury (43–48). During the harvest of the donor heart, two types of ischemia, cold and warm, follow each other. Cold ischemia occurs during the cold preservation time after hypothermic perfusion is instituted with the cessation of the donor’s circulation. Then the heart is removed from the storage container. Warm ischemia occurs during the time interval between taking the organ out of cold storage and re-establishing warm reperfusion. Transplantation involves rewarming of the donor heart and re-establishment of its coronary circulation. This is accompanied by the release of cytotoxic products of metabolism and the formation of free oxygen radicals (FORs) which produce reperfusion injury and augment the immunogenic properties of the graft. (37, 43–46) While unclear, the observed CMC AMI injury might be due to these complex pathophysiological occurrences during graft ischemia and reperfusion; thus, it could be a reliable marker for severe PGD.

Primary graft dysfunction is a complex clinical phenomenon, often without an identifiable cause and with complex pathophysiological pathways still not fully understood. While the overall occurrence varies between 3.8 and 7.4%, the overall mortality in those with PGD after OHT remains alarmingly high at up to 31.8% (9, 35, 49–52). The identified risk factors for PGD occurrence are listed in the 2014 ISHLT consensus document (9), but a reduction of these risk factors is very limited in clinical reality. The management of severe PGD remains a challenge and, per definition, includes the necessity of MCS support other than an intra-aortic balloon pump. In our center, all patients with severe PGD and hard outcomes included in this study were supported with ECMO; most received delayed therapy after OHT. While postoperative ECMO support was observed in both groups, it was more frequent in those with AMI in their biopsies (82% of patients in the AMI group vs. 32% in the non-AMI group). However, our study does not assess the success of ECMO therapy.

While early mortality after OHT is commonly dominated by PGD and multi-organ failure (MOF), late mortality is often dominated by rejection, CAV, infection, and malignancy (39, 49), similarly presented in our study cohort. In addition, our data show a high correlation between AMI and PGD, with a negative predictive value of 80% for the absence of AMI in severe PGD patients. AMI had high sensitivity and specificity of 75% and 74% for severe PGD, respectively. This correlates with our findings; patients who died within the first 90 days after transplant had a significantly higher incidence of AMI (88.9% vs. 20.7%; *P* < 0.0001) and severe PGD (77.8% vs. 20.7%; *P* = 0.0002). This is relevant because it suggests a co-dependant correlation between AMI and PGD.

The duration of survival to death or re-transplantation in the AMI group amounted to just 1.5 months, whereas that time was well over three years in the non-AMI group. This significant difference is clinically relevant because the presence of AMI in EMB might help identify those patients with unfavorable outcomes or early death. The non-AMI group had a prolonged survival, where patients most frequently died of non-cardiac-related causes, concurring with the existing results (9, 39, 49).

When the clinical outcomes were compared to further pathological observations, we did not observe a significant difference between the AMI and non-AMI groups for pAMR 1/2 and 2R/3R ACR occurrence. However, a higher occurrence of pAMR 1/2 was noted in the AMI group, which also occurred much earlier after OHT than in the non-AMI group, but without significant evidence. Our patients are routinely checked for rejection through EMBs. In the event of rejection, the immunosuppressive regimen is adapted accordingly. While we investigated the worst documented rejection in each patient, we did not investigate the immunosuppressive treatment alterations each patient received. This is important because while an aggressive immunosuppressive treatment aims to prevent rejection, it inherently increases the risk for treatment-related side effects, including long-term effects such as cancer.

CAV is one of the common causes of late death and a major limiting factor for long-term graft survival (5–8). It was observed in 20% of those without AMI, with median time to either death or re-transplantation in those patients at median 23.4 months after OHT. Pathophysiologically, it is a progressive occlusion of arteries and veins of the transplanted heart with the involvement of both epicardial and intramyocardial vessels (53, 54). It commonly remains clinically silent because of the denervation of the transplanted heart and tends to be diagnosed at an advanced stage of the disease. Presentations of CAV include myocardial infarction, congestive heart failure, arrhythmia, and/or sudden cardiac death (53–59). Because of the serious sequelae of CAV, extensive investigation has focused on risk factors, prediction, and prevention. Nevertheless, the pathogenesis is not fully understood, and the management of CAV continues to pose a challenge. However, both immune and non-immune factors in the donor and recipient have been identified as related to the development of CAV (53–59). In addition, several biomarkers in blood and tissue are found to correlate with the presence of CAV, and that may be able to predict CAV (53, 54, 59). Recent evidence suggests that novel imaging techniques have high sensitivity and specificity for detection of CAV, such as intravascular ultrasound (IVUS), optical coherence tomography (OCT) and coronary computed tomography angiography (CCTA) (55–58), but these are not yet routinely used. Efforts are ongoing to identify changes in EMB that can be predictive of the development of CAV, but further studies are needed (54). Since CAV occurred in only five non-AMI patients, no conclusions can be made about the impact and correlation with other investigated pathological observations due to the small event rate and sample size.

## Limitations

Our study is subject to the inherent limitations of observational research, which include a small sample size and low event rates for some variables. We could not adjust for confounding factors due to the same reasons. Hence, as generally true in observational research, our results do not support causal inferences or conclusions but should be interpreted in terms of associations. The double-blinded principle of histopathological evaluations reduced informational and selection bias as well as the type-I error by two experienced pathologists (LMB and MMM) without prior knowledge of the clinical or prior pathological diagnosis. Furthermore, patients were included based on their clinical outcomes over a long observational period, reducing selection and time bias. As only the worse rejection was included for each patient, any prior or subsequent less-severe rejections were not considered in the analysis. Importantly, all OHT recipients undergo standardized EMB sampling, irrespective of whether a rejection is suspected or not. This adds to the representability and objectivity of the data collection. Furthermore, our institution specializes in treating advanced heart failure and has a specialized medical team, which has remained largely consistent throughout the years. The healthcare providers entering data into the study database were also trained in correctly using the database.

## Conclusion

In those patients who underwent primary OHT and had a truncated postoperative course leading to either death or re-transplantation, acute CMC injury in EMB was a common pathological phenomenon, which correlated strongly with the clinical occurrence of severe PGD. The patients with observed AMI had significantly shorter survival times and a significantly higher occurrence of cardiac-related deaths. The presence of AMI necrosis in EMB biopsies may suggest a truncated course of disease in OHT recipients. Further studies are needed to investigate these findings.

## Data availability statement

The raw data supporting the conclusions of this article will be made available by the authors, without undue reservation.

## Ethics statement

This study was conducted under the principles outlined in the Declaration of Helsinki and was approved by the Institutional Review Board (HSC-MS-14-0139). Written informed consent for participation was not required for this study in accordance with the national legislation and the institutional requirements.

## Author contributions

MMM and MMi drafted and edited the manuscript and led the analysis. BZ, SN, SM, GO, MJ, RR, BK, and IG contributed to data collection, analysis, and edits of the manuscript. LB significantly edited the manuscript and supervised the work and submission. All authors agreed to be accountable for the content of the work.
